# Novel Approach to Reactive Oxygen Species in Nontransfusion-Dependent Thalassemia

**DOI:** 10.1155/2014/350432

**Published:** 2014-07-09

**Authors:** Paul I. Tyan, Amr H. Radwan, Assaad Eid, Anthony G. Haddad, David Wehbe, Ali T. Taher

**Affiliations:** ^1^Department of Physiology, Faculty of Medicine, American University of Beirut, Beirut, Lebanon; ^2^Department of Internal Medicine, American University of Beirut Medical Center, P.O. Box 11-0236, Beirut, Lebanon

## Abstract

The term Nontransfusion dependent thalassaemia (NTDT) was suggested to describe patients who had clinical manifestations that are too severe to be termed minor yet too mild to be termed major. Those patients are not entirely dependent on transfusions for survival. If left untreated, three main factors are responsible for the clinical sequelae of NTDT: ineffective erythropoiesis, chronic hemolytic anemia, and iron overload. Reactive oxygen species (ROS) generation in NTDT patients is caused by 2 major mechanisms. The first one is chronic hypoxia resulting from chronic anemia and ineffective erythropoiesis leading to mitochondrial damage and the second is iron overload also due to chronic anemia and tissue hypoxia leading to increase intestinal iron absorption in thalassemic patients. Oxidative damage by reactive oxygen species (generated by free globin chains and labile plasma iron) is believed to be one of the main contributors to cell injury, tissue damage, and hypercoagulability in patients with thalassemia. Independently increased ROS has been linked to a myriad of pathological outcomes such as leg ulcers, decreased wound healing, pulmonary hypertension, silent brain infarcts, and increased thrombosis to count a few. Interestingly many of those complications overlap with those found in NTDT patients.

## 1. Introduction to NTDT and Iron Overload

Thalassemia is an entity involving a collection of inherited diseases caused by defective or absent hemoglobin chain synthesis leading to anemia due to ineffective erythropoiesis. The severity of the disease depends on the genotype inherited [1–6]. Patients who carry the trait are often asymptomatic and continue to live a normal life, while *β*-thalassemia major patients suffer from many complications that may be ameliorated due to lifelong transfusions.

According to the WHO, the carrier rate of *β*-thalassemia is around 1.5% of the world population. It was also suggested that the incidence of individuals born with the severe form of the disease is 60,000 per year. Most of these patients are from regions around the tropical belt, including the Mediterranean, Middle East, central Asia, India, and southern China [7]. However, with the era of globalization and easier travel methods, migration is now facilitating the spread of the disease towards the Western countries.

Nontransfusion-dependent thalassemia (NTDT), as its name implies, is a term coined to describe those patients that do not require lifelong transfusions who instead may need emergent transfusions for specific clinical settings [8]. The primary forms of NTDT include *β*-thalassemia intermedia, hemoglobin E (HbE) *β*-thalassemia, and hemoglobin H disease [9]. These 3 clinical entities are the ones suggested such that reactive oxygen species are an integral player in the development of disease specific complications.

As opposed to thalassemia major, where transfusional induced iron overload is targeted towards the reticuloendothelial system and parenchyma, iron is amassed in patients with NTDT that differ, primarily occurs in hepatocytes [10–13]. The rate of iron loading is significantly different in thalassemia major ranging between 0.30 and 0.60 mg/kg/day versus 0.01 mg/kg/day in NTDT [14]. Iron overload in NTDT is a slow process; nevertheless, patients with the disease start experiencing iron-related morbidity beyond 10 years of age [14, 15]. The pattern of iron accumulation and the predilection of iron to target organs in NTDT is markedly different from transfusion-dependent thalassemia (TDT). Cardiac siderosis is of integral importance in management decisions in TDT as it is a major cause of morbidity and mortality; however, its importance is less pronounced in NTDT patients, even those with relatively elevated total body iron [16–19].

The master regulator of iron balance in humans is hepcidin, a peptide produced by the liver [20]. Hypoxia downregulates the expression of hepcidin, which leads to both increased intestinal iron absorption and increased release of recycled iron from the reticuloendothelial system [21, 22]. This in turn causes depletion of macrophage iron, relatively low levels of serum ferritin, and preferential portal and hepatocyte iron loading [13, 23].

The pathophysiology of iron loading in NTDT appears to be similar to that observed in patients with hereditary forms of hemochromatosis [13] and is different from that seen in thalassemia major where there is predilection for nontransferrin bound iron (NTBI) accumulation.

NTBI is a powerful catalyst for the formation of hydroxyl radicals from reduced forms of O_2_ [24]. Labile or “free” iron can convert relatively stable oxidants into powerful radicals. Iron concealed in proteins, as in catalytic sites of enzymes or stored in ferritin, is not exposed to oxygen radicals and cannot participate in this chemistry [25].

ROS are capable of causing oxidative damage to macromolecules leading to lipid peroxidation, oxidation of amino acid side chains (especially cysteine), formation of protein-protein crosslinks, oxidation of polypeptide backbones resulting in protein fragmentation, DNA damage, and DNA strand breaks [26, 27].

The liver, another concern, is also affected gravely in NTDT patients with the spectrum of injury ranging from fibrosis to hepatocellular carcinoma in hepatitis negative, chelation naïve NTDT patients [11, 12, 28–31]. Although NTDT is a nontransfusional disease, iron overload toxicity occurs in targeted organs that have specific complications in NTDT including pulmonary hypertension, leg ulcers, extramedullary hematopoiesis, endocrinopathies, and thromboembolic diseases.

In a recent study addressing pulmonary hypertension in thalassemia, patients with *β*-thalassemia intermedia (TI) had a 5-fold increased prevalence of pulmonary hypertension on right heart catheterization than patients with *β*-thalassemia major (5.7% versus 1.2%). Another common complication in NTDT, namely, leg ulcers, is more common in older patients with TI. The mechanism by which this complication is brought about is still unclear as some patients who are maintained on relatively low hemoglobin levels and have the same amount of fetal hemoglobin in TI patients do not develop ulcers. One explanation could be due to the fragility of the subcutaneous tissue of the skin of elderly TI patients due to reduced tissue oxygenation making healing more difficult after minimal trauma. Blood transfusions may provide some form of relief to the painful and indolent ulcers [15]. Yet another complication in NTDT is osteoporosis as a result of vitamin D deficiency and bone marrow expansion, which is quite common among TI patients [32, 33]. This may lead to bone pain and more importantly pathologic fractures.

There are several endocrine complications in patients with TI due to iron overload and anemia [15, 34]. Such complications include delayed puberty; however, fertility is usually preserved in these patients. In special clinical situations such as in pregnant women with TI, there is an increased risk of preterm delivery, intrauterine growth restriction, abortion, Cesarean section delivery, and thromboembolism [35]. A hypercoagulable state such as that seen in pregnancy warrants the need for anticoagulation in pregnant women especially if they have additional prothrombotic risk factors [36].

**Figure 1:**
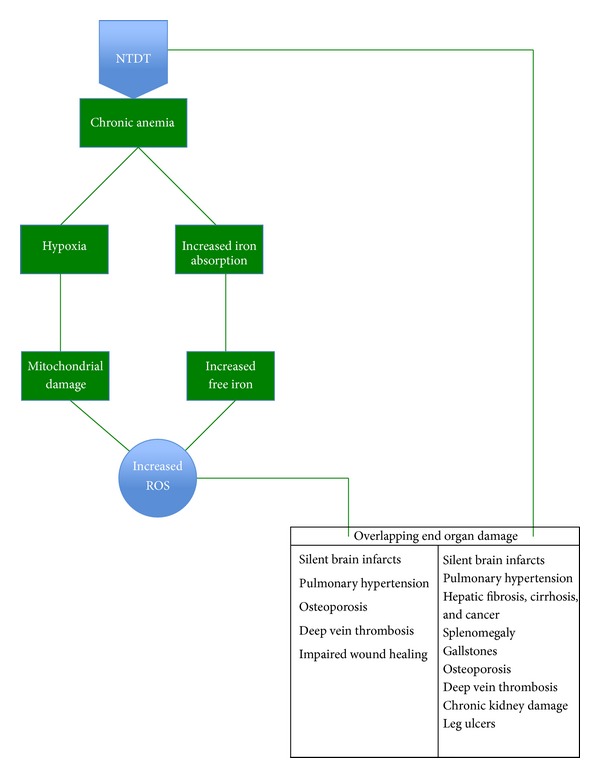
Mechanism of end organ damage in NTDT.

NTDT is associated with a hypercoagulable state, and patients with *β*-thalassemia syndromes have a pronounced risk starting childhood [37–39]. The mechanism that brings about this state of hypercoagulability in patients with NTDT is thought to be due to abnormalities in platelets along with pathological red blood cells among many other factors that are thought to contribute to clinically evident thrombotic events (Figure 1) [8, 40–44]. The largest epidemiological study to date which analyzed data from 8860 thalassemia patients (6670 thalassemia major and 2190 TI) demonstrated that thromboembolic events occurred 4.38 times more frequently in TI patients than in thalassemia major patients [45].

Renal damage is an emerging issue in TI. It is apparent that chronic hypoxia causes proximal tubular cell dysfunction and interstitial fibrosis, which, in the presence of other renal risk factors, may lead to progressive renal disease [46–48]. Early proximal tubular markers such as NAG, *β*_2_-microglobulin, phosphaturia, and uricosuria should be evaluated in these patients to detect early tubular abnormalities, which can salvage the kidney ultimately [49]. Although strokes are uncommon in TI patients, one study showed that 37.5% of patients with TI have evidence of silent brain infarction on magnetic resonance imaging (MRI) [50].

## 2. Background

Based on previously discussed data, it is clear that reactive oxygen species (ROS) are heavily implicated in the pathophysiology of NTDT. Many previous studies tested the effect of antioxidants on the treatment of NTDT. One study on fermented papaya preparation (FPP), a natural health food product obtained by biofermentation of Carica papaya, has been shown to limit oxidative stress both in vivo and in vitro [51]. Administration of FPP to patients with *β*-thalassemia major and intermedia and to patients with *β*-thalassemia/HbE disease for 3 months yielded a decrease in ROS generation, in membrane lipid peroxidation, and in externalization of phosphatidyl serine residues. There was a concomitant increase in glutathione (GSH) levels. However, no changes were observed in hematological parameters such as RBCs and hemoglobin (Hb) [52]. Curcumin, a natural herb used as food additive, contains polyphenol compounds. An extract derived from dried rhizomes of curcumin was given to patients with *β*-thalassemia/HbE disease as antioxidants [53]. It showed a decrease in iron-catalysed lipid peroxidation in vitro [54]. The results in patients treated with curcumin for one year demonstrated a significant decrease in oxidative parameters concomitant with a decrease in methemoglobin and NTBI. These changes were observed throughout the administration of curcumin. However, there were no changes in Hb levels throughout the period of treatment [55]. Vitamin E has well-established antioxidant properties. Since vitamin E is frequently deficient in homozygous *β*-thalassemia patients [56], its supplementation was studied extensively. The results showed that in heterozygotes patients, high dose of oral vitamin E decreased lipid peroxidation in RBCs and increased their survival [57]. Other studies showed improvement in the plasma antioxidant/oxidant balance, in the oxidation of low-density lipoproteins [58], and in the impaired osmotic fragility of RBCs [59]. Parenteral administration of vitamin E was more effective than oral administration [60]. Most of these studies, however, did not show a significant improvement in clinical parameters, that is, Hb concentration and transfusion requirement. All studies done on the effect of antioxidants inNTDT show a decrease in oxidative stress. However, the previous literature suggests that this decrease in oxidative stress in those patients was being tested against improvement in RBC indices. No study to date tried to link decrease ROS burden with improvement of end organ damage, in which ROS are implicated in NTDT patients.

## 3. Tissue Hypoxia and ROS

Many of the clinical manifestations of NTDT can be attributed to the chronic hypoxic environment created by the pathologic red blood cells. Another major source of ROS formation is the underlying irregular and insufficient supply of oxygen which creates a disturbed cellular physiology [61]. There is no consensus over the definition of tissue hypoxia. This is clouded further by the fact that partial oxygen pressures differ between tissues, which made many experts adopt the definition of tissue hypoxia as a condition in which the cells of a tissue have abnormal oxygen utilization such that the tissue experiences anaerobic metabolism [62]. The strongest contributor to hypoxia induced ROS is the mitochondria. In cellular hypoxia, a more reductive state is present. Reducing substances such as NADH and FADH2, which participate in the electron transport chain where oxygen is an integral part, accumulate due to the disruption in the chain. This buildup makes electrons readily available for production of ROS. Another postulated theory for the increase of mitochondrial ROS is that under hypoxic conditions nitric oxide radicals may be produced. These radicals bind and inhibit cytochrome oxidase resulting in an increased affinity towards oxygen and an increase in reduction of electron carriers upstream from the terminal oxidase. This will lead to the formation of ROS [61]. Chronic tissue hypoxia is a source of oxidative damage. This was shown by two studies, one in mice exposed to high altitude and the other by observations in hypoxic chronic obstructive pulmonary disease patients [61, 63]. The electrochemical gradient across the mitochondrial membrane (Dcm) is indicative of an active proton gradient that drives ATP synthesis [64]. The Dcm collapse observed in thalassemic patients, particularly in those who are nontransfused, shows the energetic failure under hypoxic conditions due to the metabolic switch from oxidative phosphorylation to anaerobic glycolysis. The mitochondrial impairment is coupled with endogenous ROS overproduction [65]. The hypoxia effect is highlighted by the higher lymphocytic ROS in nontransfused compared to transfused patients, despite their lower iron overload [66–68]. Redox imbalance causes an increased lipid peroxidation. Lipid peroxidation causes hemolysis, which worsens the already severe anemia and further worsens redox imbalance due to hemoglobin release [69]. Apart from hypoxia, lipid peroxidation may induce Dcm decrease with ROS overproduction, as observed in senescent cells [70] and after exposure to an environmental metal mixture [71]. Such findings strengthen the central role played by mitochondrial impairment in thalassemia. Cumulative oxidative damage, produced by iron and hypoxia, triggers a vicious cycle that may lead to organelle collapse [72].

## 4. ROS, NOX Family, and End Organ Damage

### 4.1. NOX Family

Superoxide generation by an NADPH oxidase was considered as an oddity only found in professional phagocytes. However, over the last years, six homologs of the cytochrome subunit of the phagocyte NADPH oxidase were found: NOX1, NOX3, NOX4, NOX5, DUOX1, and DUOX2.

The homologs are now referred to as the NOX family of NADPH oxidases. These enzymes share the capacity to transport electrons across the plasma membrane and to generate superoxide and other downstream ROS [72]. Of particular interest to our discussion is NOX4, which is highly expressed in the kidney, osteoclasts, endothelial cells, smooth muscle cells, hematopoietic stem cells, fibroblasts, keratinocytes, melanoma cells, and neurons. Induction of NOX4 mRNA expression is observed in response to endoplasmic reticulum stress, shear stress, hypoxia, and ischemia [73–76].

### 4.2. Oxidative Stress and Pulmonary Hypertension

**Figure 2:**
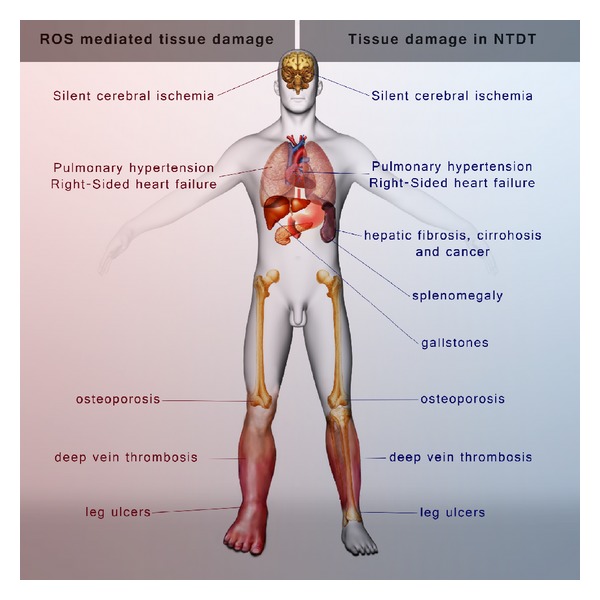
ROS mediated organ damage versus NTDT mediated tissue damage.

Nonphagocytic NADPH oxidases have recently been suggested to play a major role in the regulation of physiological and pathophysiological processes, namely, hypertrophy, remodeling, and angiogenesis in the systemic circulation. Moreover, NADPH oxidases have been suggested to serve as oxygen sensors in the lungs. Chronic hypoxia induces vascular remodeling with medial hypertrophy leading to the development of pulmonary hypertension. NOX4 has been shown to be a major player in the vascular remodeling associated with development of pulmonary hypertension [77]. Most of the available animal models of pulmonary hypertension (PHT) exhibit the two principal pathological features in the pulmonary vasculature common to most forms of PHT. These include excessive vasoconstriction and remodeling of the pulmonary arteriolar wall, primarily by a mechanism of smooth muscle proliferation within the medial layer. Because ROS may promote vasoconstriction, smooth muscle cell proliferation, and vascular remodeling, they are likely to play a critical role in many forms of PHT [78–81]. It is known that ROS can stimulate release of arachidonic acid, the substrate used for production of all arachidonic acid metabolites, including the potent constrictor, thromboxane. Therefore, ROS mediate constriction due to impaired acetylcholine (Ach) responses observed in hypoxic pulmonary arteries (Figure 2 and Table 1). This is caused by stimulating production of thromboxane [82–84].

### 4.3. Oxidative Stress and Wound Healing

Temporary hypoxia after injury triggers wound healing, but prolonged or chronic hypoxia delays wound healing. In normally healing wounds, ROS such as hydrogen peroxide (H_2_O_2_) and superoxide (O^2^) are thought to act as cellular messengers to stimulate key processes associated with wound healing, including cell motility, cytokine action, and angiogenesis. However, an increased level of ROS transcends the beneficial effect and causes additional tissue damage [85, 86]. Various damaging effects of ROS/reactive nitrogen species (RNS) can be seen in chronic wounds. An overproduction of ROS/RNS results in inactivation of epidermal enzymatic antioxidants, despite increased enzymatic antioxidant expression in the wound and significantly depletes nonenzymatic antioxidant levels in wound tissues. This results in sustained elevation and survival of ROS/RNS in chronic wounds [87]. Sustained oxidative and nitroxidative stress prolongs the inflammation in chronic wounds as both ROS and RNS stimulate neutrophil and macrophage chemotaxis and migration and also induce the expression of adhesion molecules in the capillaries. Direct cellular effects of ROS/RNS include impaired migratory, proliferative, and extracellular matrix (ECM) synthetic properties of dermal fibroblasts and keratinocytes [88]. There is excess iron deposition in the skin of patients with venous ulceration that increases the chances of free radical production by Fenton reaction [89]. There is no record to date that measures iron deposition in the skin of NTDT patients.

In summary, hypoxia stimulates wound healing such as the release of growth factors and angiogenesis [85]; however, oxidative stress is thought to play a detrimental role in the healing process.

### 4.4. Oxidative Stress and Thrombosis

The first study demonstrating that platelets were able to generate ROS was published in 1977. Currently, we know that ROS are implicated in platelet activation by (1) propagation of platelet activation by inactivating nitric oxide, (2) releasing platelet agonists such as ADP, giving formation of isoprostanes and ox-LDL, and (3) releasing proatherogenic molecules such as CD40L, which are mainly produced by NADPH oxidase [90]. ROS formation is functionally relevant for platelet activation; the role of NADPH oxidase was also studied in a model of thrombus formation upon blood perfusion at high shear. Platelet thrombus formation in samples where NADPH oxidase inhibitors were added was significantly reduced [91]. NADPH oxidase activation is also implicated in platelet mediated LDL oxidation [89]. Generally, ROS have been suggested to act as second messengers in platelet activation. Specific proposed functions of NOX-derived ROS in platelets include regulation of platelet aggregation, adhesion, and recruitment [92–96].

### 4.5. Oxidative Stress, Osteoporosis, and Brain Infarcts

NOX1 appears to be required for the differentiation of precursor into mature osteoclasts in response to the receptor activator of the NFkB ligand RANKL [97]. Experiments indicate that both NOX4 and NOX2 participate in bone resorption by activating mature osteoclasts [98]. NOX also plays a role in the CNS where stroke size was markedly reduced in NOX2-deficient mice, while increased NOX2 expression in diabetic rats was associated with an aggravated ischemic brain injury [99, 100].

## 5. Conclusion

Increased ROS formation is already proven in TDT patients. It is more pronounced in NTDT patients due to their chronic hypoxemic state resulting from less blood transfusions and a different pattern of iron accumulation that results in “free iron” ready for generating oxidative material. ROS excess has been linked to numerous pathological processes most of which coincide with those present in NTDT patients.

## Figures and Tables

**Table 1:** Mechanism of ROS damage in specific complications.

Pulmonary hypertension (PHT)	Chronic hypoxia-vascular remodeling with medial hypertrophy due to NADPH oxidases

Delayed wound healing	(i) ROS/reactive nitrogen species (RNS) overproduction prolongs the inflammation in chronic wounds as both ROS and RNS stimulate neutrophil and macrophage chemotaxis and migration (ii) Direct cellular effects of ROS/RNS include impaired migratory, proliferative and extracellular matrix (ECM) synthetic properties of dermal fibroblasts, and keratinocytes

Thrombosis	(i) Propagation of platelet activation by inactivating nitric oxide (ii) Release of platelet agonists such as ADP, giving formation of isoprostanes and ox-LDL causing the release of proatherogenic molecules such as CD40L which are mainly produced by NADPH oxidase

Osteoporosis	NOX1, NOX2, and NOX4 (NOX family of NADPH oxidases) play role in bone resorption due to activation of mature osteoclasts

Silent brain infarcts	NOX2 imbalance causes brain injury/stroke
